# Using theory to improve low back pain care in Australian Aboriginal primary care: a mixed method single cohort pilot study

**DOI:** 10.1186/s12875-016-0441-z

**Published:** 2016-04-12

**Authors:** Ivan B. Lin, Juli Coffin, Peter B. O’Sullivan

**Affiliations:** WA Centre for Rural Health, University of Western Australia, PO Box 109, Geraldton, 6531 Western Australia; Geraldton Regional Aboriginal Medical Service, PO Box 4109, Rangeway, 6531 Western Australia; Telethon Kids Institute, PO Box 855, West Perth, 6872 Western Australia; School of Physiotherapy, Curtin University, GPO Box U1987, Perth, 6845 Western Australia

**Keywords:** Research translation, Musculoskeletal pain, Quality improvement, Guidelines, Health care, Evidence based practice, Theoretical domains framework

## Abstract

**Background:**

Low back pain (LBP) care is frequently discordant with research evidence. This pilot study evaluated changes in LBP care following a systematic, theory informed intervention in a rural Australian Aboriginal Health Service. We aimed to improve three aspects of care; reduce inappropriate LBP radiological imaging referrals, increase psychosocial oriented patient assessment and, increase the provision of LBP self-management information to patients.

**Methods:**

Three interventions to improve care were developed using a four-step systematic implementation approach. A mixed methods pre/post cohort design evaluated changes in the three behaviours using a clinical audit of LBP care in a six month period prior to the intervention and then following implementation. In-depth interviews elicited the perspectives of involved General Practitioners (GPs). Qualitative analysis was guided by the theoretical domains framework.

**Results:**

The proportion of patients who received guideline inconsistent imaging referrals (GICI) improved from 4.1 GICI per 10 patients to 0.4 (95 % CI for decrease in rate: 1.6 to 5.6) amongst GPs involved in the intervention. Amongst non-participating GPs (locum/part-time GPs who commenced post-interventions) the rate of GICI increased from 1.5 to 4.4 GICI per 10 patients (95 % CI for increase in rate: .5 to 5.3). There was a modest increase in the number of patients who received LBP self-management information from participating GPs and no substantial changes to psychosocial oriented patient assessments by any participants; however GPs qualitatively reported that their behaviours had changed. Knowledge and beliefs about consequences were important behavioural domains related to changes. Environmental and resource factors including protocols for locum staff and clinical tools embedded in patient management software were future strategies identified.

**Conclusions:**

A systematic intervention model resulted in partial improvements in LBP care. Determinants of practice change amongst GPs were increased knowledge of clinical guidelines, education delivered by someone considered a trusted source of information, and awareness of the negative consequences of inappropriate practices, especially radiological imaging on patient outcomes. Inconsistent and non-evidence based practices amongst locum GPs was an issue that emerged and will be a significant future challenge. The systematic approach utilised is applicable to other services interested in improving LBP care.

## Background

The gap between evidence and practice is the one of the “most important challenges for public health in this century” p. 1 [1]. In the case of low back pain (LBP) there is increasing awareness that the tremendous burden of LBP would be reduced if health care was more concordant with evidence [2, 3]. Three significant evidence-practice gaps are inappropriate radiological imaging for LBP, addressing the psychosocial aspects of the pain experience, and providing patients with evidence based information [4, 5]. Current guidelines suggest that radiological imaging for LBP such as x-rays, Computerised Tomography (CT) or Magnetic Resonance Imaging (MRI) should only be ordered when there is suspicion of serious (e.g. cancer, fracture) or a specific pathology (severe or progressive neurological deficits), or the patient is a candidate for interventions such as surgery [6]. However, between 25–50 % of people with LBP receive an x-ray [7, 8] and this unwarranted imaging is costly, exposes the patient to radiation unnecessarily and may make patients worse by increasing their worry and inducing fear avoidance behaviours when common structural changes are reported without adequate explanation [6, 9, 10]. Psychosocial issues are amongst the strongest predictors of outcome in LBP [11]. Despite this, psychological distress, including anxiety and depression, is poorly recognised and then enacted upon by practitioners [4, 8]. A critical element of LBP care is providing patients with information that encourages self-management such as keeping physically active [12]. Despite this only 20 % of Australian patients with LBP are advised to remain active and avoid bed rest [13]. Gaps between evidence and practice such as these results in increased disability, burden, and cost.

Many different interventions have been applied to reduce evidence-practice gaps, but there is no consensus on what is most effective. Educational workshops directed toward practitioners are a common strategy. Psychosocial oriented educational workshops are reported to improve practitioner beliefs and self-reported behaviours [14, 15]. However the effect on actual practice is unclear, and is reported to have no impact or only modest improvements [16–18]. A Cochrane review that investigated interventions to improve the appropriateness of LBP radiological imaging concluded that dissemination of educational materials or clinical guidelines to clinicians resulted in minimal changes [19]. The effect of audit and feedback, whereby practitioners are provided with feedback about the number of imaging requests for LBP, is variable with some studies reporting no influence on imaging referral practices and others reporting improvements [19]. Across a broad array of health conditions, audit and feedback has been reported to result in small but potentially important improvements to professional practice, and is most effective when there are low levels of performance at baseline and when verbal and written feedback is provided [20]. Alternatively attaching education messages to lumbar spine radiological reports is reported to reduce imaging requests by doctors by 20 % [21]. ‘Passive strategies’ such as disseminating guidelines to practitioners is reported to result in modest improvements to their beliefs and intentions to practice in an evidence-based way measured via self-report questionnaire [22]. However a study that measured practitioner behaviours from patient records demonstrated poorer results [23]. Therefore more research is needed to understand which interventions, in which settings and with which populations are effective in improving the quality of LBP care.

### Systematic research translation approaches

Research translation is a rapidly developing field concerned with the adoption of evidence into practice and addressing gaps so that there is improved quality of patient care [24]. The uptake of new behaviours by practitioners is greater if research translation efforts are done in a systematic manner and based on evidence [24]. Systematic research translation approaches are useful because they provide a framework in which evidence-practice gaps are identified and the required change delineated. Further, the context is understood including the barriers and enablers to changing practices, interventions are developed that address barriers and enablers, changes are measured, and the determinants of change understood. A recent systematic review compared systematic tailored research translation strategies to untailored interventions in 15 randomised controlled trials, concluding that tailored approaches were more likely to improve practice [25]. Despite this few studies have attempted to address LBP research translation systematically [26]. Recently a randomised controlled trial, the IMPLEMENT study, applied a systematic approach to plan and implement a complex research translation intervention to improve LBP care in primary care [5]. The study reported no change in the primary outcome, LBP imaging rates, and only modest changes in practitioner intentions to practice in a manner consistent with guidelines. One potential explanation noted by the authors was that they did not address biomedical/structural beliefs about LBP held by General Practitioners (GPs) which may have been a barrier to change [5]. This issue was unaddressed in the IMPLEMENT study and suggests that there may be other determinants of LBP practice in primary care that are yet to be discovered/addressed in implementation research. As noted by French et al, further work is needed to build a cumulative evidence base in LBP implementation research [5].

This study aimed to contribute to the growing body of research in applied LBP research translation by documenting and evaluating our application of theory-based interventions in an, as yet, unreported context. The study focus was one rural Australian Aboriginal primary health care service. Primary care is a critical area to address improvements in LBP care, and rural Aboriginal primary care is arguably a priority concern due to the high burden of disease in rural and Aboriginal populations and reduced levels of support available to rural practitioners [27]. We believed that by applying a systematic evidence informed approach we could improve the quality of LBP management. We anticipated that by documenting implementation processes and outcomes, our findings could be useful to health care practitioners who are interested in improving the quality of care of the service in which they work.

## Methods

This was a single cohort pragmatic mixed methods pilot study. Mixed methods approaches can produce greater insights than quantitative or qualitative methods alone [28] and health service case studies such as this, in which a problem is studied in depth, are useful in exploring complex, multi-faceted health issues in real life settings [29].

### Setting and participants

The setting was a rural Australian Aboriginal Medical Service (AMS). Aboriginal Medical Services are the main provider of primary health care services to Aboriginal Australians [30]. Services are governed by community boards and hence promote self-determination of Aboriginal people [30]. The AMS employed over 70 full-time and part-time staff including GPs, nurses, Aboriginal Health Workers, physiotherapist, social workers, psychologist, midwives, mental health workers and staff working in health promotion programs, such as smoking cessation.

At the beginning of the project there were six fulltime GPs who saw the majority of patients with LBP, and two part-time GPs (one day per week). Because part-time GPs saw fewer patients and were unable to attend some of the interventions (e.g. educational workshops) we focussed on the behaviours of the fulltime GPs (“participating GPs”). However during the project two fulltime GPs left unexpectedly and one GP took an extended period of leave, returning prior to the end of the project. As a result four different locum GPs were employed on a short term basis (between 2 and 4 weeks) during the project after implementation interventions had been delivered. IL was the part-time physiotherapist at the AMS.

### Interventions

**Fig. 1:**
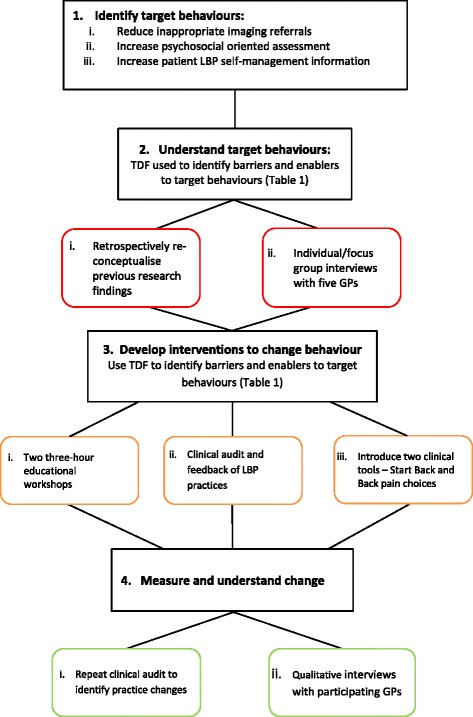
Implementation strategy involving four steps

We used a systematic implementation strategy that involved four steps (Fig. 1) [26, 31]:

1. Identifying the target behaviours. Three behaviours were identified from our previous research [9] and LBP clinical guideline recommendations [12]:
  i. reduce the number of inappropriate LBP radiological imaging referrals,
  ii. increase the proportion of patients with LBP who receive a psychosocial oriented assessment,
  iii. increase the proportion of patients receiving information encouraging LBP self-management, such as advice to keep functionally active and engage in healthy lifestyle behaviours such as regular physical activity.
2. Understanding the target behaviours. The theoretical domains framework (TDF) [32] was used to identify barriers and enablers to the three target behaviours. The TDF is a synthesis of multitude behaviour change theories into a single framework that allows assessment and explanation of health related behaviours. It consists of 14 behavioural domains and 84 constructs (Table 1). The TDF was used retrospectively to re-conceptualise our previous research findings [9, 33] and prospectively as a framework in informal qualitative interviews with GPs exploring their perceptions about the target behaviours. For example in previous qualitative research involving Aboriginal people with LBP and health practitioners such as GPs and physiotherapists, we found that Aboriginal people who were more disabled held structural/biomedical views about the cause of LBP [9]. Health practitioners acknowledged the influence of emotional factors on LBP (e.g. depression) but not cognitive influences such as beliefs about the cause or future perceptions about pain [34]. Most health practitioners believed that the underlying cause of chronic LBP (defined as LBP persisting for longer than three months) was anatomical structural failure [34]. Using the TDF as a framework we inferred that GP management behaviours, such as radiological imaging, arose due to a dominant structural/anatomical orientation toward LBP and a lack of knowledge about a biopsychosocial model of LBP or of radiological imaging guidelines. Our inferences were supported by informal interviews undertaken by IL with five GPs (one focus group interview and one individual interview) about their attitudes toward the target behaviours. In these interviews the TDF was used prospectively as a framework for GPs about their attitudes and beliefs toward the target behaviours. For example in the domain, skills & beliefs about capabilities, GPs were asked “how difficult is it to investigate and manage psychosocial factors including patient beliefs”. Interview data was recorded in the form of field notes. Qualitative thematic analysis of this data was combined with the re-conceptualisation of our previous research. Barriers were identified in seven TDF domains including; knowledge, physical skills, beliefs about consequences, social influences, memory, attention & decision processes, environmental context & resources, social professional role/identity. Enablers were identified in the TDF domains of memory, attention & decision processes, environmental context & resources, social professional role/identity, intentions, and reinforcement (Table 2).

**Table 1:** Twelve domains of the Theoretical Domains Framework (TDF), reproduced with permission from Phillips et al. [50]

TDF domain	Description
Knowledge	An awareness of the existence of something
Skills	An ability or proficiency acquired through practice
Social/professional role and identity	A coherent set of behaviors and displayed personal qualities of an individual in a social or work setting
Beliefs about capabilities	Acceptance of the truth, reality, or validity about an ability, talent, or facility that a person can put to constructive use
Optimism	The confidence that things will happen for the best, or that desired goals will be attained
Beliefs about consequences	Acceptance of the truth, reality, or validity about outcomes of a behavior in a given situation
Reinforcement	Increasing the probability of a response by arranging a dependent relationship, or contingency, between the response and a given stimulus
Intentions	A conscious decision to perform a behavior or a resolve to act in a certain way
Goals	Mental representation of outcomes or end states that an individual wants to achieve
Memory, attention and decision processes	The ability to retain information, focus selectively on aspects of the environment, and choose between two or more alternatives
Environmental context and resources	Any circumstance of a person’s situation or environment that discourage or encourage the development of skills and abilities, independence, social competence, and adaptive behavior
Social influences	Those interpersonal process that can cause an individual to change their thoughts, feelings, or behaviors
Emotion	A complex reaction pattern, involving experiential, behavioral, and physiological elements, by which the individual attempts to deal with a personally significant matter or event
Behavioral regulation	Anything aimed at managing or changing objectively observed or measured actions

**Table 2:** Analysis of barriers/enablers, TDF domain, and corresponding interventions to change the three target behaviours

Barrier/enabler	TDF domain	Intervention
Imaging for LBP		
GPs have a structural/anatomical orientation to LBP and belief that radiological imaging is useful for management	Knowledge	Educational workshops • Include biopsychosocial model of LBP (including HCP and patient beliefs) and epidemiology of LBP and imaging findings
There is limited knowledge of LBP imaging guidelines	Knowledge	Education workshops • Include LBP Imaging guideline recommendations and use of clinical tools
GPs are unsure how to advise patients that imaging is not needed	Physical Skills	Education workshops • Include skills rehearsal - patient explanation and advice
GPs do not believe there are negative consequences of unwarranted imaging	Beliefs about consequences	Education workshops • Include consequences of inappropriate imaging
There is a perception that patients expect to be investigated with imaging	Social influences	Develop appropriate patient information resource • Information scenarios where imaging is discouraged/not needed
Having imaging guidelines available will aid memory	Memory, attention & decision processes	Introduce clinical tool – LBP management • Introduce LBP decision making tool that includes imaging recommendations
Having imaging guidelines accessible are useful	Environmental context & resources	Introduce clinical tool – LBP management • Introduce LBP decision making tool that includes imaging recommendations
There is a senior GP who is “on board” and a potential opinion leader	Social professional role/identity	Education workshops • Encourage GP leader to ‘have a voice’ during workshops
Undertake biopsychosocial assessment		
There is limited understanding of the biopsychosocial model of LBP	Knowledge	Education workshops • Discuss biopsychosocial model of LBP
GPs lack skills in undertaking b-p-s assessment	Physical Skills	Education workshops • Include skills rehearsal - questions during b-p-s assessment • Explain use of clinical tools
There is inadequate time in a GP consult to undertake a b-p-s assessment	Environmental context & resources	Introduce clinical tool – b-p-s screening tool
Clinical tools can aid assist GPs remember to assess biopsychosocial factors	Memory, attention & decision processes	Introduce clinical tool – b-p-s screening tool
Provide patient information		
Most GPs would like to provide information however there is no patient LBP information available appropriate to the client group	Environmental context & resources	Develop appropriate patient information resource
Not all GPs know what to advise patients	Knowledge	Education workshops • Patient information • Explain patient information resource
Overall enablers to facilitating change in LBP care		
LBP is seen as a challenging condition to manage and staff are motivated to improve care	Intentions	Education workshops • Acknowledge and reinforce staff motivation to improve care
There is a culture within the organisation of improving practice	Social professional role/identity	Align program with other quality improvement initiatives
Educational program that accrue CPD points are valued	Reinforcement	Accredit educational workshops for CPD points with professional organisations.
The clinic has an integrated patient records system that could host tools to improve practice	Environmental context & resources	Introduce clinical tools that align with integrated patient records system
3. Developing and delivering interventions to influence practice. Three interventions were identified and developed by the authors that addressed barriers and enablers identified within different domains of the TDF (Table 2) and that had some theoretical support for addressing the desired behaviour changes [35]. Hence there was coherence between the target behaviours, barriers and enablers to enacting these behaviours, and the interventions. The interventions were:
  i. Two three hour interactive educational workshops - to address the domains of knowledge, skills, beliefs about consequences, social/professional role and identity, and intentions. The workshops were accredited by the Royal Australian College of General Practitioners so they attracted Continuing Professional Development points. This addressed the domain of reinforcement.
  ii. An audit and feedback of LBP practice – addressing the domain of intentions and social professional role/identity. A retrospective clinical audit was undertaken by IL (described below in Evaluation - Practice Outcomes). A summary of results was presented and provided to GPs at one of their regular weekly meetings. The evidence for audit and feedback in improving LBP care is inconclusive [19] however there is positive support in influencing practitioner behaviours in a broad array of health conditions [20]. In addition the AMS had utilised audit and feedback as an intervention previously to influence practices in other health conditions.
  iii. Introduction of two clinical tools; a LBP decision making tool designed for primary care [36] and the STart Back tool – a biopsychosocial prognostic risk screening tool [37]. This was used to address memory, attention and decision processes, and environmental context and resources.
    Interventions were delivered February to April 2013. Similar interventions had been used within the AMS previously to improve care for other health care conditions e.g. diabetes, however to date there had been no focus on LBP care nor had interventions been developed using a similar systematic process.
    In addition to the three interventions delivered we planned to develop culturally appropriate LBP information for Aboriginal patients [38] to address the perception that patients expect to be investigated with imaging (TDF domain - social influences) and as a patient resource for GPs (TDF domain - environmental context & resources) (Table 2). However this was not completed in sufficient time and we were unable to include it as an intervention.

4. Measuring and understanding change – collecting quantitative data on practice outcomes and qualitative data on GP perspectives relating to change (see below).

### Ethics

Ethical approval was granted by the Western Australian Aboriginal Health Ethics Committee and University of Western Australia Human Research Ethics Committee.

### Evaluation

#### Practice outcomes

Practice outcomes related to the three target behaviours were gathered via a clinical audit. All patients presenting with LBP were identified in a six month period before the project began (July-December 2011) and for a period after the intervention implementation (July-December 2013). Patients included those who had sought care for LBP defined as pain, muscle tension, or stiffness localized below the costal margin and above the inferior gluteal folds, with or without leg pain and of short or long term duration. We excluded ‘red flag’ conditions (e.g. facture, tumour, inflammatory disorders) or referred pain of diagnosable non-spinal origin (e.g. visceral referred pain). Guidelines recommend that, in the absence of red flags conditions or pain of non-spinal origin, radiological imaging is used prudently, psychosocial influences are considered, and self-management strategies such as keeping active are recommended [12, 36]. Patients were identified through search of Communicare (™Telstra Health), the electronic clinical records management system used by the AMS. Communicare records were searched for all diagnosis codes that related to LBP (e.g. back pain chronic, acute low back pain, lumbar pain, lumbago, chronic back pain), all referrals for radiological imaging for LBP (x-ray, CT and MRI), and all referrals from GPs to e.g. specialists, physiotherapy, for a LBP related complaint. Patient records were then manually reviewed to assess whether they were in accordance with the inclusion criteria. Radiological imaging referrals and patient records were reviewed and imaging referral behaviour were classified as guideline consistent or guideline inconsistent according to a synopsis of radiological imaging guidelines [36]. The presence of a psychosocial assessment was recorded as “yes” if there were any indications from patient records that psychological, cognitive or social factors were included as part of patient assessment [39]. This included the use of the screening tools that included psychosocial factors such as the STart Back [37]. The provision of LBP information that encouraged self-management was recorded as “yes” if there were any indications from patient records, for example patients had been advised or given information encouraging them to stay functionally active and/or engage in healthy lifestyle such as regular physical activity. The audit was undertaken by IL.

#### Qualitative GP perspectives

At the conclusion of the project four participating GPs were interviewed to ascertain their perspectives about LBP care and the change process. Interviews were conducted by IL in March 2014, one year following the interventions. This was chosen because it was toward the end of the project and GPs would have the opportunity to reflect on their practices some time following the interventions. In –depth interviews elicited GP’s views on the three target behaviours. The TDF was used as the theoretical framework to guide discussions. GPs were also prompted to reflect on the intervention strategies employed, the changes that had occurred in the clinical audit and sustainability – how to maintain/facilitate high quality of LBP care into the future. An unanticipated change during the project was a greater number of locum GPs employed and so how to ensure consistency of practice amongst short-term locum staff was also discussed.

### Analysis

Quantitative practice outcomes data was entered into Statistical Package for the Social Sciences (IBM, Version 21). The number of guideline inconsistent imaging referrals (GICI), psychosocially orientated assessments undertaken, and LBP information provided was calculated as a proportion of the total number of LBP patients attending (evaluated separately for participating and non-participating GPs) over each of the two six month periods (pre- and during intervention), and expressed as a rate per 10 LBP patients. Differences in the rates between the pre-intervention and intervention rate were calculated with accompanying 95 % confidence intervals.

In-depth interviews with GPs were transcribed and entered into NVivo (QSR International, Version 10). Following repeated re-reading of the data deductive thematic analysis was undertaken using the TDF as the analysis framework [32]. Statements relating to each of the target behaviours were coded against the TDF domains. Under each relevant domain the statements were coded as a inhibiting or facilitating each of the target behaviours [40]. Aspects not included within the TDF framework e.g. strategies employed, changes, and sustainability were analysed inductively. Initial analysis was conducted by IL and findings were discussed amongst authors to critically examine the relationship between the data and preliminary results. A psychologist who was experienced in research translation, familiar with use of the TDF and external to the project reviewed the TDF domains, themes and coded statements to assess coherence. Findings were then refined following discussion and re-examination of the data. The result was an interpretive description that considered the knowledge all investigators brought to the study as well as the findings from the quantitative data [41].

## Results

### Practice outcomes

#### Imaging

Participating GPs were consulted by 44 LBP patients in the pre-intervention period, 18 of which were referred for imaging inconsistently with guidelines (4.1 GICI per 10 pts) (Table 3). In the intervention period the same GPs were consulted by 46 LBP patients, only 2 of which were referred for imaging inconsistently with guidelines (0.4 GICI per 10 pts), a decrease in the rate of GICI of 3.7 GICI per 10 patients from pre to post interventions (95 % CI for decrease in rate: 1.6 to 5.6).

**Table 3:** Practice behaviours of GPs

	Jul-Dec 2011	Jul-Dec 2013	95 % CI for change in GICI per 10 LBP pts
GPs - participated in the intervention			
LBP patients	44	46	
Imaging referrals - guideline inconsistent (rate per 10 LBP pts)	18 (4.1)	2 (.4)	1.6 to 5.6
Psychosocial assessment undertaken (rate per 10 LBP pts)	3 (.7)	5 (1.1)	−1.6 to 0.8
LBP information provided (rate per 10 LBP pts)	9 (2.0)	17 (3.7)	−3.8 to 5.6
GPs who did not participate - part-time/locum staff			
LBP patients	33	41	
Imaging referrals - GICI (rate per 10 LBP pts)	5 (1.5)	18 (4.4)	−5.3 to -.5
Psychosocial assessment undertaken (rate per 10 LBP pts)	2 (.6)	3 (.7)	−1.3 to 1.1
LBP information provided (rate per 10 LBP pts)	10 (3.0)	8 (2.0)	−1.2 to 3.4

Non-participating GPs, including those working part-time and as a locum, were consulted by 33 patients in the pre-intervention period of which 5 were referred for imaging inconsistently with guideline recommendations (1.5 GICI per 10 pts) (Table 3). In the intervention period 41 patients were seen by non-participating GPs and 18 referred for imaging that was inconsistent with guidelines (4.4 GICI per 10 pts), representing an increase in GICI of 2.9 GICI per 10 patients from pre to post interventions (95 % CI for increase in rate: .5 to 5.3).

#### Psychosocial oriented assessment

For participating GPs the number of psychosocial oriented assessments increased slightly from 3 to 5 assessments, an increase of .4 assessments per 10 patients from pre to post interventions (95 % CI for change in rate: 1.6 decrease to 0.8 increase). For non-participating GPs there were 2 psychosocial oriented assessments in the pre-intervention period and 3 in the intervention period, a difference of .1 per 10 patients (95 % CI for change in rate: 1.3 decrease to 1.1 increase) (Table 3).

There was no recorded use of the STart Back tool by GPs within patient records.

#### Self-management information

For participating GPs there was an increase in the rate of patient self-management information provided from 2 to 3.17 per 10 patients from pre to post interventions (95 % CI for change in rate: 3.8 decrease to 5.6 increase) (Table 3). Amongst non-participating GPs there was a reduction in the rate of self-management information provided from 3 to 2 per 10 patients (95 % CI for change: 1.2 decrease to 3.4 increase) (Table 3).

### GP perspectives

#### Changes to practice

General Practitioners described a number of positive changes in each of the three target behaviours despite that the audit results found no changes to psychosocial assessments or the provision of LBP self-management information (Table 4). Most GPs felt that they had changed their practices, however these were often not recorded in patient records (Table 4). Sometimes this was because of time constraints, or that there was not a system to record these practices easily in the patient record software.

**Table 4:** Changes to practice and recording in patient records

Imaging:
*“I know for a fact I haven’t ordered a single back x-ray.”* (Participant 2)
Psychosocial assessment:
*“I suspect, this is what I think, that a lot of the GPs are doing psychosocial assessments, but we don’t have a way of recording it automatically. I think people just talk about it, you know?”* (Participant 4)
*“..we start screening that psychological assessment. So that’s changed. The last time we usually don’t do that because that’s sometimes never even come into your mind, just go for medical model, maybe physical, psychological assessment lacking*”. (Participant 1)
*“..discussing mental health issues, and what is the barrier for them, like not going for physio, like what are their thoughts or beliefs, like about the pain and the progress of the disability*” (Participant 3)
Self-management information:
*“we started discussing more about how to take care of back pain, and how - what are the strategies which can help them, they started - things have changed, really” (*Participant 3)
*“Give them pamphlets; give them that educational material which you very kindly gave us on back ache”* (Participant 4)
Recording in patient records *“..typing into the case note is not a priority because we’ve got a time of 20 min and then under the pressure of the workflow. So we - because not everyone is good at typing as well. So they probably have to type into the more significant medically related things. But it’s come into the last, right, sometimes you didn’t even type it at all*.” (Participant 1)

#### Determinants of change

The determinants of change were identified including enablers and barriers to practices that had occurred during the project, and with regard to changing practices in the future (Table 5). Enablers were identified in seven domains of the TDF (knowledge, beliefs about consequences, environment context resources, goals, social professional role, social influences, behavioural regulation) and barriers in two domains (environment context resources, social influences).

**Table 5:** Determinants of change – enablers and barriers with major TDF domains, themes and illustrative quotes

TDF domain	Theme	Illustrative quote
Enablers		
Knowledge	Changes to knowledge	*“…then we are not going for radiology until the red flag signs are there which are really serious indicators for radiology or something. So we’re giving more importance to conservative management and not jumping on radiology or medical treatment.”* (Participant 1)
		*“…(managing patients) discussing mental health issues, and what is the barrier for them, like not going for physio, like what are their thoughts or beliefs, like about the pain and the progress of the disability.”* (Participant 3)
Knowledge	Changes for new staff	*“..I think either you do it or we do it (educational workshops), every year with our new doctors make sure they have access to the information and the training so that they know why this is the way we do it (manage low back pain).”* (Participant 2)
Beliefs about consequences	Imaging	*“..now I understand that until the red flags signs or something really needs to be - management is going to change, then I’m not referring patients that much (for imaging), and I’m doing management by ourselves here…. (Previously) if an unnecessary patient was going to see a specialist and there was not going to be any change in the management, then a few patients were getting unnecessary radiology.”* (Participant 3)
		*“Trying to wean them off imaging, because imaging really puts a negative scenario, “oh, I’ve got something wrong with my back and it can’t be cured”.* (Participant 4)
Environment context resources	Teamwork on site	*“if you want the guidelines you need a supported team, otherwise it doesn’t really help the patient and they don’t feel like we are doing good enough and they rely on medical model”* (Participant 1)
Environment context resources	Patient resources/Communication	*“So we have to come up with a way of being able to explain that in, probably, a written way, a speaking way, maybe a video way; maybe a group way of trying to explain what chronic pain is and what that perception is and why we use this multi-modality. Until we can do that and we can communicate that well, we are stuck with a group of people who are absolutely sure that every time they move their back in a certain way they are injuring their back.”* (Participant 2)
Environment context resources	Funding model	*“Given that we can offer them (patient) the facilities - not every doctor can offer them facilities. They have to - I mean, we have the - now, we have that they get these things relatively - not an out of pocket expense. I think that’s a very important factor as well.* (Participant 4)
Environment context resources	Processes for locum staff	*“… for the sleepers or [other] medication we ask locums also to follow strictly....protocol, so maybe for back pain also, or radiology….we put it on everyone’s clinic, maybe good not to do unnecessary radiological investigations. Because you can’t specifically advise them to do that, but in general if we are putting something like that (protocol), that may be good.”* (Participant 2)
Goals	Holistic practice	*“…I think we’ve given ourselves enough time to do it (biopsychosocial LBP assessment), and we consider it a priority for dealing with, I guess, the multi-morbidity of our patients.”* (Participant 2)
Social professional role	GP role	*“…we are the first point of encounter. So if we can do a bit of (best practice) more on the first encounter that will be easier for everyone to support (The patient)”* (Participant 1)
Social influences	Trust in investigator	*“Before we had guidelines…. and those things, but we were not following that much. But when you showed us videos and case discussions and those things, then we realised that, yeah, the things are really important, how we deal with patients.*” (Participant 3)
Behavioural regulation	Audit and feedback	*“I think another area where you get behavioural change is if you regularly audit and you provide feedback….So looking at whether people are using it and whether it’s changing their practice and what sort of feedback they’re getting from it allows, I guess, you to look at where it falls apart.”* (Participant 2)
Barriers		
TDF domain	Theme	Illustrative quote
Environment context resources – barrier	Locum staff	*“If locums came in and they looked at - and they did what we did, it would not be a problem, but we - the trouble with locums is that they quite frequently have their own way of doing things. They come in and they don’t tend to really work with what’s going on, because it’s all just too hard for them to learn, I guess. I don’t know. It may be something about the personality of people who do locums.”* (Participant 2)
Environment context resources – barrier	Clinical tools/recording practices	*“(The STart Back) needs to be on the computer somewhere where you can get it. This is the problem with online tools. There’s no way of recording whether you’ve used them so it needs to be made into an interactive whatever that can be used on Communicare and becomes a document on their file. You create a document. We’ve got them for mini-mentals, we’ve got them for other tools*” (Participant 2)
Environment context resources – barrier	Teamwork availability	*“..from my side, I think it’s a bit of a hassle, because we have the psychologist here only two and a half days, two days a week. There is some waiting list for them to see a psychologist. He’s also busy with really the mental health issues, rather than chronic pain issues.”* (Participant 4)
Social influences – barrier	Other doctors	*“…it’s very hard when they’ve (the patient has) already seen somebody else and they’ve already been told a bunch of information and got a whole bunch of expectations or whatever. I think I’ve really, really, really struggled to get people to move beyond when that’s been their attitude.”* (Participant 2)
Social influences – barrier	Workers compensation	“*Because they - some employer organisations they are - they’re told that without the x-ray evidence or whatever they really don’t want to help [the patient] back to their job.”* (Participant 1)

Enablers for changes that occurred during the project included increased knowledge about when and when not to refer for radiological imaging, the psychosocial aspects of LBP, and the importance of providing information to patients. Similarly there was greater awareness of the consequences of unwarranted imaging – including patients being steered unnecessarily down a ‘medicalised pathway’. Participating GPs viewed the three target behaviours as part of their professional role and they shared a goal to improve care and provide ‘holistic practice’ to patients. Improving care was enabled by the context of the service with an interprofessional team available onsite, although the availability of some team members, such as a psychologist, was sometimes a barrier. The principal investigator (IL) was a seen as positive social influence on GPs because he was viewed as a trusted source of knowledge. Audit and feedback was seen to be a useful way to encourage behaviour change and was familiar to participants as it had been used for a variety of quality improvement programs.

A significant contextual barrier was the practices of locum staff, of which there had been an increase in number during the implementation stage of the project. GPs felt that the practices of locum staff were very varied and hard to influence because of their entrenched practice behaviours, the short term nature of their appointments, their “personality”, and they operated outside usual clinic processes. Suggestions of how the health service context could influence the practices of locums included; ensuring there was continual staffing so locums weren’t needed, and developing orientation processes and protocols for locums to follow during LBP care. However overall, the practices of locum staff were viewed as a significant challenge.

The online evidence based clinical tools implemented in the project were separate to the usual clinic software and GPs found this a barrier to their use. GPs preferred clinical tools to be integrated into the usual clinical software and in a format that was saved automatically into patient records. Most GPs indicated that more patients had received psychosocially oriented assessments and LBP self-information than what was recorded in patient records, and that a “tick box” in patient records would be an easier way for them to record when these behaviours occur. GPs suggested adapting the tools into a user friendly format that was integrated with clinic software, as opposed to an external website.

Other barriers related to the negative social influence of other doctors who do not practice in an evidence based manner, and the impact this had on the beliefs and expectations of patients. Patients who had a work related injury was also viewed as a barrier because GPs felt that there was an expectation by insurance companies that patients who had injured themself at work should receive radiological imaging for a low back injury.

## Discussion

Our aim, to improve LBP care in an Australian Aboriginal Primary Health Service provided by GPs in this pilot investigation, was partially achieved. There was a reduction in inappropriate imaging referrals amongst participating GPs. There were no changes in psychosocially oriented LBP assessments or the provision of LBP information; although in qualitative interviews GPs suggested that they were performing more of these practice behaviours than was being recorded. However an unforeseen issue was the practices of locum GPs.

Guideline inconsistent LBP imaging referrals amongst participating GPs reduced from 18/44 patients to 2/46 patients during the project. The primary interventions used to influence this behaviour (and TDF behavioural domain) were three educational workshops. The determinants of change were; improved knowledge of LBP imaging guidelines (knowledge), an awareness of the negative consequences of inappropriate imaging (beliefs about consequences), and education delivered by a trusted source (social influence). In our initial analysis we identified the availability of clinical tools, including imaging guidelines (environmental context and resources) and practitioner skills in explaining to patients why imaging was not needed (skills) however these were less prominent determinants of practice change. GPs continued to identify the expectations of insurance companies for imaging, and to a lesser degree patients, as social influences. A novel approach in our study was that in education workshops for GPs we focussed on the negative consequences of inappropriate radiological imaging, in particular the potential negative effects on patient beliefs and iatrogenic LBP disability [9]. Other studies, including the IMPLEMENT trial have focussed on the consequences of missing an underlying pathology by not imaging, radiation exposure, cost, or for imaging to guide treatment [26, 40]. Our findings suggest that to improve LBP imaging referral practices, educational workshops should focus on evidence based recommendations for radiological imaging, practitioner knowledge and beliefs about the consequences in relation to the potential negative effects of imaging to the patient within a biopsychosocial model of LBP, and delivered by someone who is identified as a trusted source of information.

There were no changes to psychosocial oriented assessments reflected in audit results. However GPs indicated qualitatively that they understood more about psychosocial factors (knowledge) and were providing more psychosocially oriented assessments than was reflected by clinical audit data. In our study we implemented the STarT Back tool [37] to aid memory, attention and decision processes for GPs to deliver psychosocially oriented care. GPs felt the STarT Back was useful however there was poor uptake/recording. On reflection, to improve the uptake of STarT Back and recording of psychosocial care we needed greater attention to the environment context. This includes embedding STarT Back within the clinic management software or introducing a “tick box” within electronic patient records when psychosocial assessment and care has been provided. As has been noted with other online clinical aids, to increase uptake tools need to be integrated into existing systems and routine workflow [42]. This would also allow reliable recording of practice for future research.

There was a modest increase in the provision of self-management information amongst participating and GPs indicated qualitatively that they were providing more self-management advice to patients than was reflected in audit results. The potential mismatch between GP reports and self-management advice measured via clinical audit means that future work needs to develop a more reliable system for recording self-management advice that is integrated into usual clinical systems [5]. One planned intervention to increase the delivery of LBP self-management information was the development of culturally appropriate LBP information. This was not developed in time for GPs to utilise however GPs did make use of a printed patient information leaflet provided during one education session. This suggests that the availability of patient information resources, in addition to knowledge of what information to provide to patients, is again, a key environmental and contextual enabler to this behaviour.

A significant unforeseen issue that emerged during the project were the practices of locum GPs (environment context & resources) that was not identified in our initial analysis. During the project, imaging rates increased from 5/33 guideline inconsistent imaging referrals per patient to 18/44 for locum/part-time staff with no changes in psychosocially oriented LBP assessments or the provision of LBP information. During informal discussion about this study, one locum GP revealed to IL how he considered himself experienced in musculoskeletal medicine and was confident managing patients with LBP. Influencing the practices of short term locum staff will, without doubt, be a substantial challenge, and more research examining the beliefs and attitudes of this group about LBP and the determinants of their practices would be valuable. Research has highlighted the challenges of maintaining the quality of care in the rural context where there are higher numbers of locum staff and greater workforce turnover [43, 44] however little research has reported interventions aiming to improve the quality of care in this context. Participating GPs were very aware of this issue, and identified environmental and contextual factors, including better orientation processes and protocols for LBP care and focussing on consistent staffing levels, as priorities. Other strategies may need to be at a systems level within the organisation, such as professional leadership and focussing on a ‘culture’ of evidence based practice [45]. More broadly, influencing medical practices in LBP care such as during medical training, or via well-resourced social marketing campaigns [46] may be needed.

The gap between LBP evidence and practice is a tremendous issue facing health care systems and systematic approaches to improve care are needed. Systematic LBP research translation is in its infancy. Our pilot study contributes by describing the process used to understand, plan and implement change in one primary care service. Our interventions resulted in improvements in two of our desired behaviours. A recent trial, the IMPLEMENT study, resulted in modest improvements in practitioner intentions to practice in an evidence based manner and no change in measured behaviour; lumbar spine imaging referrals [5]. Both our study and the IMPLEMENT study used a systematic, step-by-step approach including the TDF. Comparing both studies must be done with caution as they are different in their scope and context, and observational research may have inflated outcomes compared to experimental studies. Nevertheless there is value in contrasting the findings and discussing the implications for future LBP implementation endeavours.

Firstly, we found improvements in imaging behaviours and self-management advice amongst participating GPs to be generally coherent with our analysis of barriers and enablers and the interventions we developed in response to these, providing support for this approach. However although this approach has theoretical support, to date there is a dearth of research and hence little evidence that there are superior better outcomes in LBP care when a systematic, theory directed approach is taken [5]. More research utilising systematic, theory-informed approaches are needed. Secondly, in our behavioural analysis, with the exception of intentions and reinforcement, we identified the same behavioural domains as the IMPLEMENT study (knowledge, physical skills, beliefs about consequences, social influences, memory, attention and decision processes, environmental context & resources, social professional role/identity, and reinforcement) but different barriers and enablers. For example as previously discussed, we identified the domain “beliefs about consequences” with respect to GPs who were unaware of the negative consequence of inappropriate imaging practices on patient outcomes, whereas this was not a primary focus in the IMPLEMENT study [26]. Differences in the barriers and enablers between the two studies may be because of the different study contexts or different methods employed to investigate barriers and enablers. The authors of IMPLEMENT postulated that a lack of change in GPs behaviours may have been due to a biomedical or structural orientation toward LBP [5], suggesting that this was a barrier that may have been missed in their initial behavioural analysis. In contrast this was a barrier we identified and aimed to address in our interventions. Both studies used qualitative methods in the analysis of behaviour however different findings may reflect a different perspective of researchers. Improving the identification of modifiable barriers to LBP practice is important if they are to be addressed. We suggest that LBP implementation researchers who are targeting similar behaviours consider the barriers and enablers that our study, the IMPLEMENT trial [26] and other studies have identified [40, 47] and examine these factors for relevancy within their populations. We anticipate that as the field of LBP implementation research develops the modifiable determinants of LBP behaviours will become more refined (e.g. knowledge, memory) and their applicability for which target populations in which contexts understood so as to guide practice improvement initiatives.

Lastly our study differed significantly to the IMPLEMENT study because it was a led a by service ‘insider’. Data suggests that IL’s position within the health service may have been a positive influence on outcomes. Project ‘champions’ can improve implementation success by increasing motivation and act as a positive social influence for change within their organisation [24]. However it may be difficult to identify and engage change champions in a large multisite study. Also, intensive systematic approaches such as in our study are resource intensive, intellectually and financially [48], and may be challenging to replicate across a number of settings.

This was a pilot investigation and there is a tension between smaller service level implementation initiatives and the need to scale-up interventions so that practice improvements may be applied across broader populations. Our findings suggest several avenues that could contribute to the issue of broader change and that warrant further exploration. Firstly and as we have discussed, while the outcomes of practitioner education on improving LBP radiological imaging referral practices is equivocal [19], we recommend education that incorporates the negative consequences of inappropriate imaging on patient outcomes [9]. Secondly, reducing environmental contextual barriers to evidence based practice by integrating evidence based tools into usual clinical systems warrants investigation. In our study a future priority is the STarT Back tool. Electronic clinical decision making tools integrated into usual care processes have been trialled successfully in other areas of primary care [49] however the potential application to LBP care is in its infancy.

The strengths of this pilot project were the application of a careful systematic approach and the use of quantitative and qualitative methods to provide an in-depth analysis. The limitations are that it is in one health care service involving a small number of GP’s and the transferability of the findings elsewhere are unknown. One planned intervention strategy was the development of culturally appropriate LBP information (Lin et al- submitted) which was aiming to increase the amount of patients receiving LBP information. Unfortunately this was not developed and tested in a time to include within the project and is the focus of ongoing work. Another potential factor was that IL was a service ‘insider’ and responsible for implementation of interventions and data collection, potentially introducing the element of bias. We attempted to minimise bias by having clear criteria for the clinical audits and in the qualitative analysis by ensuring there are steps to ensure rigour. Although during interviews participants were very forthcoming, it is possible that IL’s role may have influenced disclosure or the perspectives of interviewees, including viewing IL as a “trusted source of information”. Alternatively it is also possible that, as an insider, IL was also uniquely positioned to understand the context of the service, and this may have positively influenced disclosure. Other methods exist to understand the determinants of change, for example to quantitatively measure participants knowledge, skills and beliefs about their capabilities to undertake the desired behaviours before and following the interventions. This could be considered in future work to compliment qualitative perspectives. There was a two year time period for the project and it is possible that other factors external to the project may be responsible for changes. One activity was a one-day interprofessional symposium for health practitioners coordinated by IL and PO, and that also included a pain medicine specialist and clinical psychologist. The symposium focussed on assessing and managing persistent pain conditions and held in the town in which the study was conducted. There were no known state or national campaigns targeting better LBP care. It is possible that the interprofessional symposium may have reinforced information presented to GP participants within the educational workshops as the content was very similar and thus contributed to change. However although this is possible, we believe it is unlikely to have been a major influence as participants discussed project interventions during interviews, linking these to changes. A significant advantage of the approach is that it has provided insight into the determinants of change, and therefore enabled us to prioritise areas to address in the future improvement of care.

## Conclusion

The application of a systematic research translation approach resulted in partial improvements in LBP care. Determinants of practice change amongst GPs in relation to radiological imaging referrals were increased knowledge of clinical guidelines, education delivered by someone considered a trusted source of information, and awareness of the negative consequences of inappropriate practices, especially radiological imaging. The effect on other behaviours was less clear. We have identified issues that we need to address to improve LBP care further; the practices of locum staff and improved integration of clinical tools into clinical software are two priorities. Our approach and findings are applicable to other health services interested in improving LBP care and contributes to the cumulative evidence base in LBP implementation research.

## Abbreviations

AMS: Aboriginal Medical Service
GICI: guideline inconsistent imaging referrals
GP: General Practitioner
LBP: low back pain
TDF: theoretical domains framework
